# Electrocution Stigmas in Organ Damage: The Pathological Marks

**DOI:** 10.3390/diagnostics11040682

**Published:** 2021-04-10

**Authors:** Gelsomina Mansueto, Mario Di Napoli, Pasquale Mascolo, Anna Carfora, Pierluca Zangani, Bruno Della Pietra, Carlo Pietro Campobasso

**Affiliations:** 1 Department of Advanced Medical and Surgical Sciences, University of Campania “Luigi Vanvitelli”, 80138 Naples, Italy; 2 Clinical Department of Laboratory Services and Public Health—Legal Medicine Unit, University of Campania “Luigi Vanvitelli”, via Luciano Armanni 5, 80138 Naples, Italy; pasquale.mascolo@unicampania.it (P.M.); pierluca.zangani@unicampania.it (P.Z.); bruno.dellapietra@unicampania.it (B.D.P.); carlopietro.campobasso@unicampania.it (C.P.C.); 3 Neurological Service, SS Annunziata Hospital, Viale Mazzini 100, 67039 L’Aquila, Italy; mariodinapoli@katamail.com; 4 Department of Experimental Medicine Legal Medicine Unit, University of Campania “Luigi Vanvitelli”, via Luciano Armanni 5, 80138 Naples, Italy; anna.carfora@unicampania.it

**Keywords:** autopsy, histology, electrocution, heart, brain, lung, skin, rhabdomyolysis

## Abstract

Background: Diagnostic criteria for electrocution related death are still a challenge in forensic pathology and it seems that the electrical mark is the only reliable evidence. Methods: A comparison of histological and morphological findings of skin and internal organs from an autopsy series of electrocution deaths with those mostly reported in literature as representative for electrocution. Results: The morphological changes of heart, brain and other main internal organs are still unspecific. Organ’s damage observed in electrocution deaths shows a wide variability, not reliable for a certain diagnosis of electrocution. The electrical mark is still the golden standard for diagnosis of electrocution. Conclusions: In electrocution related deaths, pathological findings of the main internal organs are not enough evidence to support with certainty a post-mortem diagnosis that a victim suffered an electrical damage. Although the organ histological changes are undoubtedly the starting point for a better understanding of the fatal even, the diagnosis of death from electrical damage is still a dark and unsolved chapter. The electrical mark still represents a fundamental indicator above all in the medical-legal field, but the identification of pathognomonic elements and signs not limited to the skin alone could be a valid help in the future, especially in unclear cases.

## 1. Introduction

In the United States, there are approximately 30,000 shock incidents annually with 1000 deaths because of electrical injuries [1,2]. The patient with electrical damage is difficult to manage clinically especially due to the scarce literature and guidelines. Electrocution related death remains a dark scenario and requires numerous and valid signs for the correct medico-legal diagnosis. The electric burn is undoubtedly the most evident macroscopic appearance representing the starting hypothesis but it can also be misleading in the classification of death [3,4,5,6,7,8]. What are the aspects to consider in the absence of electric burn for a correct diagnosis? How should the different electrical marks be observed in the context of the documentary data for the possible exclusion of comorbidities in causal relationship with death? There are no well-defined criteria in the literature to follow for the evaluation of organs damage from electrocution. Some authors describe the electrical mark both macroscopically and microscopically, but in a few papers attention is paid to the search for organ morphological change in the hope that these may be ever more numerous and useful in the diagnosis of death. However, it seems that the electrical mark is the most sought after and validating sign.

Since the victim’s examination can prove negative, a proper electrocution related death investigation requires a high level of suspicion with careful documentation of the death scene and the autopsy findings. Furthermore, it must be considered that in case of electrocution due to direct (DC) or alternating current (AC) at high voltage burns and non-specific findings of asphyxiation have been also reported [9]. The victims of low voltage AC often do not show electrical burns in the absence of characteristic findings of ventricular fibrillation. Low-voltage rarely produces death. The causes of death from electrocution are often ventricular fibrillation rather than atrial fibrillation and paralysis of the central respiratory control center and respiratory muscles [10]. Finally, the alterations of blood coagulation and circulation induced by electrical damage must also be considered. The morphological changes of heart, brain and other organs are still unclear. Few studies report unequivocal and irrefutable organ signs in deaths from electrical damage at high voltage and even less at low voltage. Finally, since electrocution death could also be confused with sudden death, in forensic pathology literature several authors suggest to examine the pathological findings in the various organ for a correct differential diagnosis [11,12,13,14,15].

This review attempts to summarize existing evidence of electrical damage in main organs that seems a relatively neglect topic and to define the main types of tissue electrical injuries and their role in the mechanism of death.

### Pathophysiology of Electrocution Damage

The set of pathophysiological events resulting from the action of an electric current on the human body are defined as an electric shock. The term ‘electrocution’ defines the electrical injuries that result in death. Mainly, injuries due to electricity occur by three mechanisms: (1) the direct effect of electrical current on body tissues; (b) the conversion of electrical energy to thermal energy, resulting in deep and superficial burns; (c) mechanical injury due to muscle contraction, or as a complication of a fall after electrocution. The primary injury determinant is the current amount that flows through the body and the site of contact. The effects of increasing current intensities on the body are shown in Table 1. Further factors able to the extent of injury are the voltage, resistance, type of current (AC or DC), the current pathway, and duration of contact [16]. The main tissue damage determined by most electrical currents is primarily due to the thermal energy (or heat) generated by the current, according by Joul’s law:

(1) $$P=I^{2}\times R\times t$$

where *P* is heat evoked per seconds (expressed units of watts, or joules per second), *I* is current intensity (Amperes), *R* is resistance (Ohms) and *t* is the time of contact (in seconds). The resistance is a function of the area of contact, pressure applied, and the presence of moisture. The Ohm’s law I=V/R expresses the relationship among these factors. From this physical law, higher resistance tissues have a tendency to heat up and coagulate, (i.e., skin, bone, and fat) while tissue with lower resistances (i.e., nerves and blood vessels) transmit current. Between all soft tissues, the skin can show the most severe effects of an electrical injury. Dry skin has a resistance of approximately 100,000 ohms; but only less than 2500 ohms when the skin is dampened [17].

Thus, in some cases, a lower voltage applied to tissue with low resistance can generate more current and be more damaging than higher voltage applied to tissue with high resistance. In addition, the type of current can produce different effects: while DC current tends to cause a single muscle spasm that throws the victim from the source, on the contrary AC repetitively stimulates muscle contraction. Because frequently, the site of exposure is at the hand, and the flexors of the arm are stronger than the extensors, the victim grasping the source prolongs the duration of contact perpetuating tissue injury. Furthermore, the amount of AC needed to cause injury varies in proportion to its frequency (Hz). For example, an electric stimulus with frequencies between 15 and 150 Hz produce a tetanic skeletal muscle contraction with a current intensity of 20 mA current while this intensity may not be perceptible at 10 Hz. However, the same current may cause respiratory paralysis or ventricular fibrillation at lower frequencies [19]. An electrical injury can damage both the central and peripheral nervous systems. Manifestations may include loss of consciousness, weakness or paralysis, respiratory depression, autonomic dysfunction, and memory disturbances [20,21,22,23]. The involvement of peripheral nervous system is common with both sensory and motor findings. Lower-extremity weakness may go undiagnosed initially especially if not searched and the patient attempts to ambulate. Interestingly, the sensory deficits usually do not correspond to the motor findings with a patchy distribution. Furthermore, high-voltage exposures can show their clinical manifestations of neurologic damage delayed for days to months after the injury. One specific aspect of lightning injuries is keraunoparalysis or Charcot’s paralysis that is a temporary paralysis characterized by blue, mottled, and pulseless extremities (lower more commonly than upper). These findings are felt to be secondary to vascular spasm and often resolve within hours but can be permanent [22,23]. Patients hit by lightning may present with pupils that are fixed and dilated or asymmetric due to autonomic dysfunction. As a result, fixed, dilated, or asymmetric pupils should not be used as a reason to stop resuscitation.

## 2. Materials and Methods

In order to address the electrocution pathophysiology with the main organ changes and possible differential diagnoses, a comparison of the literature data with histopathological data of an autopsy series of electrocution death has been performed. A review of the pertinent literature related to electrocution and life-threatening electrical injuries has been made by searching English-language publications listed in PubMed using the following search terms electrocution, electrical mark, and high voltage, low voltage, electrical mark, lung damage, heart damage, brain damage, kidney damage, rhabdomyolysis, fractures, death, autopsy, histology. Reference lists from identified articles were also screened. Articles included relevant retrospective studies, case reports, pathological cases and series, and review articles published between 1978 and 2020.

## 3. Results and Discussion (Main Histological Findings and Differential Diagnosis)

960 articles were totally reviewed. However, only 16 papers satisfied the inclusion criteria reporting histological finding of organ lesions apart from the skin were considered. These are reported in Table 2 along the distribution of main histological findings. The main morphological findings observed in the internal organs are summarized as follows.

### 3.1. Skin Damage

**Figure 1:**
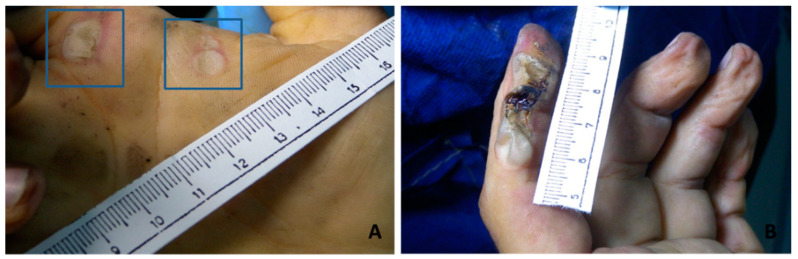
Macroscopic findings. Electrical lesions on the right palm (**A** insert) from low voltage; electric lesion of the dorsal face of the second finger on the left hand from high voltage (**B**).

**Figure 2:**
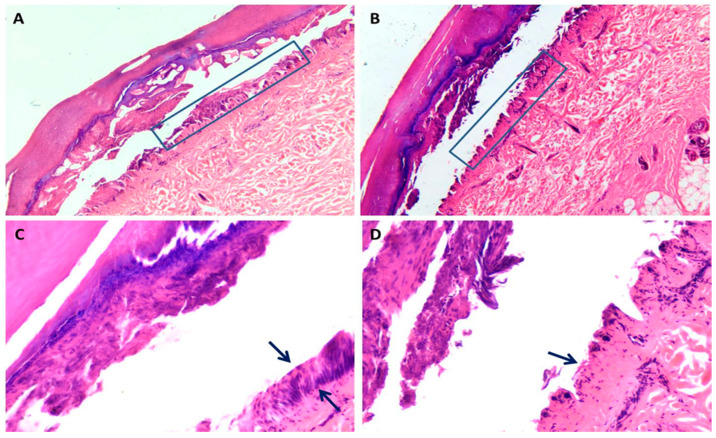
Skin histology from low and high voltage electrocution. (**A**–**D**): comparison of two cases of low (**A**–**C**) and high voltage (**B**–**D**) electrocution respectively with preserved basal epithelial layer in (**A**) (box indicates the detail) and dermal-epidermal detachment loss in (**B**) (box indicates the detail) (H&E stain ×20). In (**C**) black arrow indicates the presence of epidermal basal layer (H&E stain × 40), while in (**D**) loss of basal epidermal layer (H&E stain × 40).

The dermal-epidermal or suprabasal epidermal detachment that can even affect the most superficial layers of the epidermis is the main histological finding of an electrical damage. Separation in the epidermis along with the formation of microvesicles and separation of the epidermis from dermal papillae are common findings in electrical marks [27]. This detachment can be more or less continuous; cytologically it is characterized by cellular stretching, microvesicles, nuclear elongation with hyperchromasia and/or pyknosis with collagen-like modifications of the superficial dermis is mainly produced by high voltage current [27,28,37]. For a correct histological diagnosis it is necessary to distinguish other lesions with similar morphological aspects such as abrasions, fire lesions, etc. Flame burns and abrasions that can occur in some blunt traumas have some histological features similar to lesions caused by electrocution [24]. For example, the rate of coagulative necrosis in the epidermis and intraepidermal detachment are more evident and frequent in electrical lesions while elongation of the epithelial cell nuclei and dermo-epidermal detachment may be present in flame burn lesions or abrasions but with different degrees. However, the severity of nuclear elongation and coagulative necrosis due to heat and current flow have been found to be highly significant in electrical lesions as well as dermal-epidermal detachment is extended to the whole lesion and continuous in high voltage (HV) electrocution [24].The most interesting and useful data correlate the different skin lesions to different voltages. In fact, no subepidermal detachment was observed in skin exposed to 4, 12, 18, 24 and 36 V, but varying degrees of detachment were observed in the samples exposed to 48 V and above 48 V [27]. It would appear that epidermal detachment is predominantly supra-basal with less large areas in low voltage exposures as opposed to high voltage exposures in which detachment appears larger and predominantly dermo-epidermal. Finally, it seems that the most important morphological histological aspect is the dermal homogenization and collaginization-like behavior especially in HV electrocution [8,14,24]. Figure 1 shows macroscopic apparency of electric burn from low and high voltage electrocution. Figure 2 shows some microscopic findings of low and high voltage skin lesions taken from our autopsy series.

### 3.2. Central Nervous System Damage

**Figure 3:**
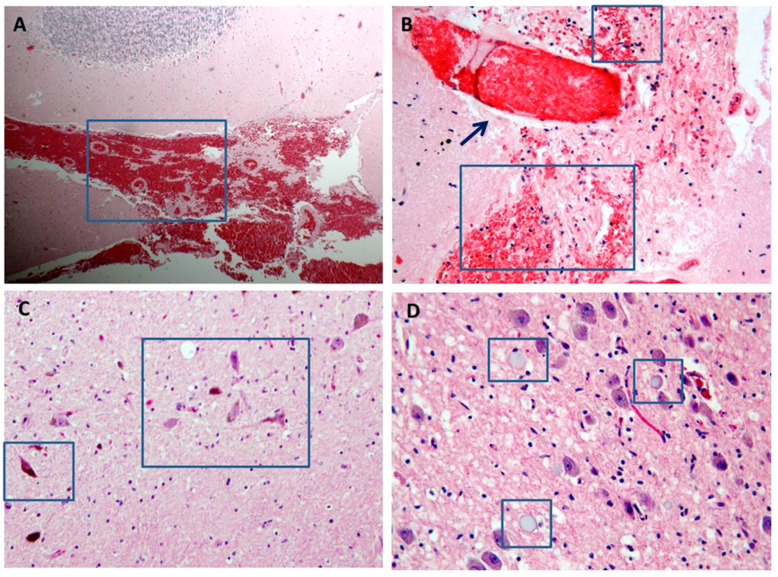
Brain histology from high voltage electrocution death. (**A**,**B**): massive cerebellar and brain hemorrhage respectively (blue box indicates the detail) with intra-vessel coagulopathy in (**B**) (black arrow indicates the detail) (H&E stain ×10 **A**; ×20 **B**). (**C**): dark neurons as sign of ischemic neuronal damage due to vasoconstriction (H&E stain ×20). (**D**): axonal balloon degeneration (blue box indicates the detail) in a context of white matter necrosis and degeneration (H&E stain ×40).

Nervous system lesions can be primary when the current travels through the brain, or secondary. Animal studies have shown pyramidal cell loss, Purkinje cell reduction and leptomeningeal lesions, bleeding, demyelination and neuronal loss in the spinal cord [38,39]. In humans, high-voltage electrical accidents can produce acute stroke, cerebral vein thrombosis from increased blood coagulation, edema, or traumatic and non-traumatic hemorrhage from vasospasm with venous hyperemia as well as axons fragmentation, degeneration, and necrosis. Unfortunately, there are not many pathological data in the literature [9,29,31,40,41] Figure 3 shows the main morphological findings observed in our autopsy series. More accurate morphological information could be obtained with specific histological methods to study the morphology of neurons and cytoarchitectony of different brain areas (i.e., Nissl staining). Electrocution damage can result in ischemic stroke from impaired blood circulation or in hemorrhagic stroke from direct structural damage with necrosis and axonal degeneration. Sub-arachnoid hemorrhages from trauma should also be considered.

### 3.3. Cardiac Damage

**Figure 4:**
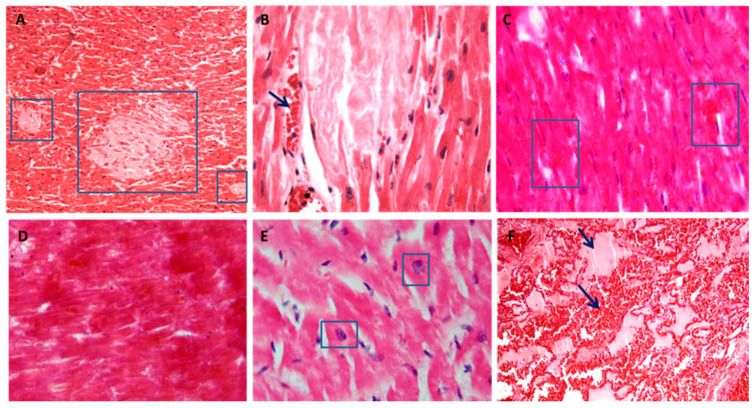
The heart and lung histology from high voltage electrocution. Different localizations of necrosis. (**A**,**B**): multiple and diffuse foci in the absence of inflammatory infiltrate (blue box indicates the detail) with erythrocyte extravasation (black arrow indicates the detail) (H&E stain ×10 in **A**, ×40 in **B**). (**C**,**D**): large areas of coagulative necrosis with loss of nuclei and with erythrocyte extravasation in (**C**) (blue box indicates the detail) (H&E stain ×10). (**E**): fragmentation of myofibers with enlarged and distorted nuclei (blue box indicate the detail) (H&E stain ×40). (**F**): pulmonary intra-alveolar edema and hemorrhage (black arrow indicates the detail) (H&E stain ×10).

The cardiac histology can be characterized from diffuse focal myocardial necrosis including specialized conducting tissue, necrosis of smooth muscle cells in the middle tunic of coronary arteries, and nuclear cytological changes. The bundle of His with its branches can be more or less affected. The neural structures of the heart are generally minimally involved [32]. More often there is a clear passage between damaged myocardium and healthy myocardium, the presence of interstitial erythrocyte and or the presence of myoglobin, that, some have also found in the kidney, as an indirect and peripheral sign. Few studies indicate a break-up of myocardial fibers with square nuclei as a possible indicator for the diagnosis of malignant arrhythmia followed by cardiac arrest from ventricular fibrillation, especially to distinguish between damage caused by high and low voltage electrocution. In larger studies, interstitial myocardial hemorrhagic infiltration is the only differentiating finding. All these aspects and, in particular, the presence of interstitial erythrocytes still require validation in the differential diagnosis between low and high voltage electrocution [33]. Myofiber break-up includes several histopathological patterns, such as, bundles of distended myocardial cells alternating with hyper-contracted cells with widening or segmentation of the intercalated discs, square nuclei (rather than the ovoid morphology) and, non-eosinophilic bands of hyper-contracted sarcomeres [34]. They should not be considered artifacts secondary to histological processing, because they can also occur in sudden deaths due cardiac arrhythmias. The histological findings of the myocardium in electrocution deaths must be careful considered since other diseases can show similar defects overlapping morphological aspects [42]. Thickness and fragmentation of cardiomyocytes with anisonucleosis, myofiber break-up or square nuclei in hypercontracted myocites are also common markers of ventricular fibrillation and ventricular hypertrophy [33,34,43]. Interstitial hemorrhagic infiltration and necrosis has been also reported in electrocution deaths as expression of direct electric damage, but they can also occur in cases of hypoxic damage. Myocardial necrosis can be the expression of direct electrical damage or vasospasm, but also of a vasospasm and other pathologies on a hypoxic basis [44]. The search for other aspects such as the presence or absence of inflammation in some cases can be useful for a differential diagnosis. Both functional and structural cardiac pathologies must be considered. The arrhythmogenic ventricular cardiomyopathy (AVC) represents a common background for an abnormal transmission of the cardiac electrical impulse, generating fatal arrhythmias [12]. Finally, when the electrical damage affects the cardiac conduction system, the fatal cardiac arrhythmia often does not allow identifying specific histopathological aspects; in this case, especially when the electrical mark is not present the sudden death entity can be confusing. In Figure 4A–E the morphological aspects observed in cases of high voltage electrocution from our autopsy series are reported. These aspects can be a guide in the diagnosis but in a coherent documentary context and considering both the antecedent state of the subject and the differential diagnoses that we have briefly treated.

### 3.4. Pulmonary Damage

In electrocution, the fibrillation with heart failure is responsible for congestive pulmonary edema. Pulmonary congestion and edema are the most common pulmonary findings and lungs are often overinflated and heavy [29,30,31,35]. However, rare cases of non-cardiogenic lung injury are described in the literature as an underdiagnosed clinical entity [45]. This entity occurs with any neurological or non-neurological event that stimulates the vasomotor centers. There are divergent theories to explain the cause of this rare phenomenon, but none have been proven. In any case, the pulmonary histological findings are characterized by diffuse and aqueous edema from hydrostatic pressure alteration between the vascular and interstitial compartments. Therefore, attention should be paid to the type and extension of pulmonary edema [35]. The distinction between functional edema and proteinaceous edema which instead occurs in diffuse alveolar damage, and the presence of endoalveolar hemorrhages are fundamental and useful for a correct differential diagnosis. The presence of hyaline membranes, elements of inflammation and/or pigmentiferous elements together with edema must be pathological aspects useful to evaluate the possibility of pre-existing and other causes of death [35]. In Figure 4F main aspects of pulmonary intra-alveolar edema and hemorrhage in high voltage electrocution death are reported.

### 3.5. Muscles Damage

**Figure 5:**
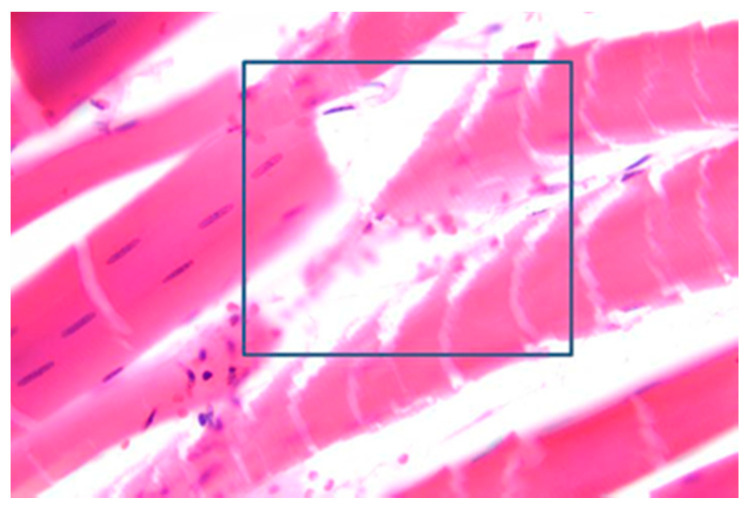
Tetanization-induced rhabdomyolysis. Fragmented striated muscle fibers with necrosis and erythrocytes (blue box indicates the detail) (H&E stain ×63).

Fracture or dislocations due to avulsion of muscles and tendons, coagulative necrosis of the soft tissues and rhabdomyolysis are the main aspects deriving from high tension [46]. Histologically, tetanization-induced rhabdomyolysis is characterized by fragmentation of muscle fibers with associated necrosis and erythrocytes and, ultimately, by acute kidney injury due to accumulation of myoglobinuria mainly in survivors [30,46]. In the differential diagnosis of sudden death it is also necessary to consider other forms of muscle and kidney damage such as rhabdomyolysis from cocaine, exertion, or epilepsy [11]. Figure 5 shows histological findings from high voltage electrocution.

## 4. Conclusions

In electrocution deaths, postmortem morphological findings are usually not evident and generally non-specific for an electrical damage except for the skin histological. Death due to electrocution is closely related to the micromorphology of electrical marks, depending on the properties of electricity, duration and site of contact [35]. Although the organ histological changes are undoubtedly, the starting point for a better understanding of the fatal even, the diagnosis of death from electrical damage is still a dark and unsolved scenario. The electrical mark and its histopathological defects still represent a fundamental indicator above all in the forensic field. The identification of pathognomonic elements and signs not limited to the skin alone could be a valid help in the future, especially in unclear cases. However, fatal electrocution may occur with no skin mark whatsoever. In these cases, the diagnosis is still entirely dependent upon the circumstances of the death [47,48].

## Figures and Tables

**Table 1:** Pathophysiologic effects of different intensities of electrical current [18].

Current Intensity	Effect
1 mA	Tingling sensation; almost not perceptible
3–5 mA	“Let-go” current for an average child
6–9 mA	“Let-go” current for an average adult
16 mA	Maximum current a person can grasp and “let go”
16–20 mA	Tetany of skeletal muscles
20–50 mA	Paralysis of respiratory muscles; respiratory arrest
50–100 mA	Threshold for ventricular fibrillation
>2 A	Asystole
15–30 A	Common household circuit breakers
240 A	Maximal intensity of household current (U.S.)

**Table 2:** Histological findings from autopsy study.

Study	Current Intensity	Skin	CNS	Heart	Lung	Other
Wang, et al. [24]	LV	Slight elongation of the epidermal cell nuclei. Detachment in corneous layer				
Akyildiz et al. [25]	N	Greater nuclear elongation in electrocution than flame burns, and abrasion				
Zhang J, et al. [26]	HV	Dermal-epidermal detachment; elongated and polarized epidermal cells with darkly nuclei				
Mondello, et al. [8]	HV	Elongated and polarized epidermal cells. Dermis coagulative necrosis				
Bellini, et al. [14]	HV/LV	Palisade nuclei in the spinous layer and lysis of the granular layer; incomplete dermo-epidermal detachment; iron and copper deposit				
Uzun, et al. [27]	HV	Coagulative necrosis of the epidermis; intra-epidermal and sub-epidermal detachment; nuclear elongation with darkly nuclei Homogenized collagen like dermis				
Sangita, et al. [28]	HV	Intra-epidermal and subepidermal detachment; epidermis coagulative necrosis; elongated and polarized epidermal cells with dark nuclei Dermal homogenization collagen like with vascular dilatation, congestion, hemorrage and thrombosis				
Visonà, et al. [29]	HV	Horny layer vacuolization, epidemic cells elongation Fragmentation and necrosis of the elastic dermal fibers	Subarachnoid hemorrhage from trauma	Hemopericardium	Hemorrhage from contusion	Visceral edema and congestion
Pfeiffer, et al. [30]	HV	Dermopidermis detachment with basal cells elongation		Fresh myocardial fiber necrosis	Hemorrhages	Kidney hemorrhage; hemoglobin detection
Wang, et al. [24]	HV			Aorta and pulmonary artery perforation (Electronic microscopy)		
Shaha, et al. [31]	N			Edema		Edema
Shetty, et al. [32]	N			Myocardium necrosis without inflammatory reaction; myocardial fragmentation and contraction bands; pericardial surface hemorrhage		
Gentile, et al. [33]	HV			Myocardial hemorrhages		
Fineschi, et al. [34].	N			Myofiber break-up; square nucleus; bundles of hyper-contracted myocites (electrocution compared with cocaine and other trauma)		
Michiue, et al. [35]	HV			Myocardial fibers fragmentation; cardiomyolysis	Hemorrhage; edema	
DeBono, et al. [36]	HV					Muscle fiber fragmentation; red blood cell extravasation; neural damage;

Abbreviations: HV indicates high voltage; LV low voltage; N no information.

## Data Availability

University of Campania L. Vanvitelli, Naples, Italy.

## References

[1] Trivedi T.K., Liu C., Antonio A.L.M., Wheaton N., Kreger V., Yap A., Schriger D., Elmore J.G. (2019). Injuries Associated With Standing Electric Scooter Use. JAMA Netw. Open.

[2] Zemaitis M.R., Foris L.A., Lopez R.A., Huecker M.R. (2021). Electrical Injuries. StatPearls.

[3] Waldmann V., Narayanan K., Combes N., Jost D., Jouven X., Marijon E. (2018). Electrical cardiac injuries: Current concepts and management. Eur. Heart J..

[4] Parakkattil J., Kandasamy S., Das S., Devnath G.P., Chaudhari V.A., Shaha K.K. (2017). Atypical Exit Wound in High-Voltage Electrocution. Am. J. Forensic Med. Pathol..

[5] Peng Z., Shikui C. (1995). Study on electrocution death by low-voltage. Forensic Sci. Int..

[6] Wright R.K. (1983). Death or Injury Caused by Electrocution. Clin. Lab. Med..

[7] Aquila I., Gratteri S., Amirante C., Fineschi V., Frati P., Ricci P. (2018). Electric or traumatic injury? The role of histopathological investigations. Med. Leg. J..

[8] Mondello C., Micali A., Cardia L., Argo A., Zerbo S., Spagnolo E.V. (2018). Forensic tools for the diagnosis of electrocution death: Case study and literature review. Med. Leg. J..

[9] Nizhu L.N., Hasan M.J., Rabbani R. (2020). High-voltage electrocution-induced pulmonary injury and cerebellar hemorrhage with fractures in atlas. Trauma Case Rep..

[10] Dechent D., Emonds T., Stunder D., Schmiedchen K., Kraus T., Driessen S. (2020). Direct current electrical injuries: A systematic review of case reports and case series. Burns.

[11] Paternoster M., Capasso E., Di Lorenzo P., Mansueto G. (2018). Fatal exertional rhabdomyolysis. Literature review and our experience in forensic thanatology. Leg. Med. (Tokyo Jpn.).

[12] Mansueto G., Benincasa G., Capasso E., Graziano V., Russo M., Niola M., Napoli C., Buccelli C. (2020). Autoptic findings of sudden cardiac death (SCD) in patients with arrhythmogenic ventricular cardiomiopathy (AVC) from left ventricle and biventricular involvement. Pathol. Res. Pract..

[13] Mansueto G., Niola M., Napoli C. (2020). Can COVID 2019 induce a specific cardiovascular damage or it exacerbates pre-existing cardiovascular diseases?. Pathol. Res. Pract..

[14] Bellini E., Gambassi G., Nucci G., Benvenuti M., Landi G., Gabbrielli M., Vanezis P. (2016). Death by electrocution: Histological technique for copper detection on the electric mark. Forensic Sci. Int..

[15] Russo C.V., Sacca F., Paternoster M., Buonomo A.R., Gentile I., Scotto R., Morra V.B., Mansueto G. (2020). Post-mortem diagnosis of invasive pulmonary aspergillosis after alemtuzumab treatment for multiple sclerosis. Mult. Scler. J..

[16] Lee R.C., Zhang D., Hannig J. (2000). Biophysical Injury Mechanisms in Electrical Shock Trauma. Annu. Rev. Biomed. Eng..

[17] Jain S., Bandi V. (1999). ELECTRICAL AND LIGHTNING INJURIES. Crit. Care Clin..

[18] Koumbourlis A.C. (2002). Electrical injuries. Crit. Care Med..

[19] Wright R.K., Davis J.H. (1980). The investigation of electrical deaths: A report of 220 fatalities. J. Forensic Sci..

[20] Ramati A., Pliskin N.H., Keedy S., Erwin R.J., Fink J.W., Bodnar E.N., Lee R.C., Cooper M.A., Kelley K., Sweeney J.A. (2009). Alteration in Functional Brain Systems After Electrical Injury. J. Neurotrauma.

[21] Cherington M. (2005). Spectrum of neurologic complications of lightning injuries. Neurorehabilition.

[22] Davis C., Engeln A., Johnson E.L., McIntosh S.E., Zafren K., Islas A.A., McStay C., Smith W.R., Cushing T. (2014). Wilderness Medical Society Practice Guidelines for the Prevention and Treatment of Lightning Injuries: 2014 Update. Wilderness Environ. Med..

[23] Davis C., Engeln A., Johnson E., McIntosh S.E., Zafren K., Islas A.A., McStay C., Smith W., Cushing T. (2012). Wilderness Medical Society Practice Guidelines for the Prevention and Treatment of Lightning Injuries. Wilderness Environ. Med..

[24] Wang T., Zou D., Zhang J., Chen Y. (2016). Application of Microbeam X-Ray Fluorescence Spectrometry in the Diagnosis of Suspected Electrocution by High-Voltage Direct Current: A Case Report. Am. J. Forensic Med. Pathol..

[25] Akyildiz E., Uzun I., Inanici M.A., Baloglu H. (2009). Computerized Image Analysis in Differentiation of Skin Lesions Caused by Electrocution, Flame Burns, and Abrasion. J. Forensic Sci..

[26] Zhang J., Lin W., Lin H., Wang Z., Dong H. (2017). Identification of Skin Electrical Injury Using Infrared Imaging: A Possible Complementary Tool for Histological Examination. PLoS ONE.

[27] Uzun I., Akyildiz E., Inanici M.A. (2008). Histopathological differentiation of skin lesions caused by electrocution, flame burns and abrasion. Forensic Sci. Int..

[28] Sangita C., Garima G., Jayanthi Y., Arneet A., Neelkamal K. (2018). Histological indicators of cutaneous lesions caused by electrocution, flame burn and impact abrasion. Med. Sci. Law.

[29] Visona S.D., Chen Y., Bernardi P., Andrello L., Osculati A. (2018). Diagnosis of electrocution: The application of scanning electron microscope and energy-dispersive X-ray spectroscopy in five cases. Forensic Sci. Int..

[30] Pfeiffer H., Du Chesne A., Brinkmann B. (2006). An unusual case of homicidal near drowning followed by electrocution. Int. J. Leg. Med..

[31] Shaha K.K., Joe A.E. (2010). Electrocution-related mortality: A retrospective review of 118 deaths in Coimbatore, India, between January 2002 and December 2006. Med. Sci. Law.

[32] Shetty B.S.K., Kanchan T., Acharya J., Naik R. (2014). Cardiac pathology in fatal electrocution. Burns.

[33] Gentile G., Andreola S., Bailo P., Boracchi M., Fociani P., Piccinini A., Zoja R. (2020). A Pilot Study on the Diagnosis of Fatal Electrocution by the Detection of Myocardial Microhemorrhages. J. Forensic Sci..

[34] Fineschi V., Karch S.B., D’Errico S., Pomara C., Riezzo I., Turillazzi E. (2006). Cardiac pathology in death from electrocution. Int. J. Leg. Med..

[35] Michiue T., Ishikawa T., Zhao D., Kamikodai Y., Zhu B.-L., Maeda H. (2009). Pathological and biochemical analysis of the pathophysiology of fatal electrocution in five autopsy cases. Leg. Med..

[36] Debono R. (1999). A histological analysis of a high voltage electric current injury to an upper limb. Burns.

[37] Blumenthal R. (2009). A retrospective descriptive study of electrocution deaths in Gauteng, South Africa: 2001–2004. Burns.

[38] Schulze C., Peters M., Baumgartner W., Wohlsein P. (2016). Electrical Injuries in Animals: Causes, Pathogenesis, and Morphological Findings. Vet. Pathol..

[39] Kurtulus A., Acar K., Adiguzel E., Boz B. (2009). Hippocampal neuron loss due to electric injury in rats: A stereological study. Leg. Med..

[40] Kokatnur L., Rudrappa M. (2016). Acute Stroke due to Electrocution: Uncommon or Unrecognized?. Case Rep. Neurol. Med..

[41] Patel A., Lo R. (1993). Electric injury with cerebral venous thrombosis. Case report and review of the literature. Stroke.

[42] Campobasso C.P., Dell’Erba A.S., Addante A., Zotti F., Marzullo A., Colonna M.F. (2008). Sudden Cardiac Death and Myocardial Ischemia Indicators: A comparative study of four immunohistochemical markers. Am. J. Forensic Med. Pathol..

[43] Baroldi G., Silver M.D., Parolini M., Pomara C., Turillazzi E., Fineschi V. (2005). Myofiberbreak-up: A marker of ventricular fibrillation in sudden cardiac death. Int. J. Cardiol..

[44] Aimo A., Di Paolo M., Castiglione V., Modena M., Barison A., Benvenuti M., Bugelli V., Campobasso C.P., Guidi B., Camici P.G. (2020). Scared to Death: Emotional Stress Causing Fatal Myocardial Infarction With Nonobstructed Coronary Arteries in Women. JACC Case Rep..

[45] Singh S., Sankar J., Dubey N. (2011). Non-cardiogenic pulmonary oedema following accidental electrocution in a toddler. BMJ Case Rep..

[46] Wang Q., Michiue T., Ishikawa T., Zhu B.-L., Maeda H. (2011). Combined analyses of creatine kinase MB, cardiac troponin I and myoglobin in pericardial and cerebrospinal fluids to investigate myocardial and skeletal muscle injury in medicolegal autopsy cases. Leg. Med..

[47] Di Maio V.J., Di Maio D. (2001). Forensic Pathology.

[48] Saukko P., Knight B. (2015). Knight’s Forensic Pathology.

